# Unveiling Hidden *Aconitum* Alkaloids in a Poisoning-Implicated Tincture by Untargeted Screening and Molecular Networking

**DOI:** 10.3390/toxins18060255

**Published:** 2026-06-05

**Authors:** Qian He, Micong Jin, Jing Zhou, Hongshun Zhang, Chengye Sun

**Affiliations:** 1 State Key Laboratory of Trauma and Chemical Poisoning, National Institute of Occupational Health and Poison Control, Chinese Center for Disease Control and Prevention, Beijing 100050, China; heqian@niohp.chinacdc.cn (Q.H.);; 2 Ningbo Key Laboratory of Coastal Environment and Health, Ningbo Municipal Center for Disease Control and Prevention, Ningbo 315010, China

**Keywords:** *Aconitum* alkaloids, pseudoaconitine, 8-deacetylpseudoaconitine, 3′-methoxyacoforestinine, untargeted screening, molecular networking, *Aconitum* poisoning

## Abstract

*Aconitum* poisoning is a major public health concern in East Asia, and remains difficult to diagnose when the causative toxins are not covered by routine targeted assays. In a poisoning incident that occurred in 2018, 15 individuals were affected, including five fatalities, after accidentally consuming a medicinal tincture during a shared meal. The comprehensive alkaloid profile of the tincture implicated in the poisoning was achieved through the integration of targeted analysis, molecular networking, and untargeted screening based on ultra-high performance liquid chromatography coupled to time-of-flight mass spectrometry, aiming to clarify the causative agents. Targeted quantitative analysis detected nine alkaloids derived from *Aconitum* plants, confirming the presence of *Aconitum* ingredients in the medicinal tincture. However, these alkaloids were either present at low concentrations or exhibited low toxicity, and thus were not the principal causative agents of this poisoning incident. Molecular networking revealed additional hidden diester-diterpenoid alkaloids (DDAs) and monoester-diterpenoid alkaloids (MDAs) that were undetected by targeted analysis. Untargeted screening identified 58 *Aconitum* alkaloids, including 15 DDAs, 17 MDAs, 17 amino-diterpenoid alkaloids (ADAs), 2 C20-diterpenoid alkaloids, and seven unclassified alkaloids. The three most abundant alkaloids were structurally identified as pseudoaconitine, 8-deacetylpseudoaconitine, and 3′-methoxyacoforestinine, and were identified as the main causative agents of this poisoning. To our knowledge, this is the first detection of these alkaloids in *Aconitum* poisoning in China. These findings demonstrate that integrated targeted and untargeted toxicological analysis can identify undocumented toxins in poisoning events of unknown origin and clarify the chemical etiology of unusual *Aconitum* poisoning.

## 1. Introduction

Poisoning caused by aconite occurs worldwide and is particularly frequent in East Asia [1,2]. In China, aconite poisoning has resulted in a substantial number of cases and fatalities, representing a significant public health concern that warrants heightened attention. Between 2004 and 2013, the Public Health Emergency Management Information System reported 49 aconite poisoning incidents involving 456 cases and 69 deaths, with a case fatality rate of 15.13% (69/456) [3]. From 2010 to 2020, the National Foodborne Disease Surveillance Network reported 380 aconite poisoning outbreaks involving 1889 cases and 75 deaths, with a case fatality rate of 3.97% (75/1889) [4]. Aconite poisoning also occurs frequently in Hong Kong [1,5,6]. A 50-year retrospective analysis (1961–2010) demonstrated that aconite poisoning constituted 16.8% of all plant food poisoning incidents in Japan, with 78 incidents, 218 patients, and 10 deaths—the highest mortality among all plant toxins [7]. Although aconite poisoning is less common in other countries and regions than in Asia, sporadic cases continue to be reported. In 2022, Canada experienced three aconite poisoning events with 14 cases across British Columbia and Ontario [8]. Cases of aconite poisoning have also been reported in Germany [9], Republic of Korea [10]. Most poisoning incidents occur when *Aconitum* is employed for therapeutic or tonic purposes, resulting from improper processing, accidental ingestion of medicated liquor intended for external use, or misidentification as other medicinal herbs [3,4]. Additionally, inadvertent collection and consumption of *Aconitum* seedlings mistaken for edible wild vegetables constitutes a notable cause [3]. Intentional poisoning and suicidal ingestion are encountered occasionally [7].

The genus *Aconitum* L. (aconite) belongs to Ranunculaceae, and comprises approximately 400 species distributed primarily in the temperate regions of the Northern Hemisphere [11,12]. China represents a major center of diversity, harboring 211 species, of which 166 are endemic [11]. *Aconitum* species exhibit extensive pharmacological activities, including cardiotonic, analgesic, and anti-inflammatory effects. Several species, notably *Aconitum carmichaelii* Debeaux and *Aconitum kusnezoffii* Reichb., have been cultivated and utilized in China for over a thousand years, with documented applications in traditional medicine dating back to ancient pharmacopeias [13,14,15]. In certain regions of China, such as parts of Yunnan and Shaanxi, *Aconitum* roots are also consumed as vegetables or food, especially in the winter or cold day [16,17]. Conversely, *Aconitum* species possess potent toxicity; inadvertent ingestion or improper use can readily lead to poisoning or even death. Aconite poisoning primarily affects the gastrointestinal, neurological, and cardiovascular systems [15,18,19]. Characterized by a short latent period and abrupt onset, clinical manifestations include perioral and limb paresthesia, nausea, vomiting, hypotension, and various types of arrhythmias. Among these, refractory electrical storm and ventricular fibrillation constitute the principal causes of mortality [2]. No specific antidote or targeted therapeutic remedy exists for *Aconitum* poisoning; patient management and clinical rescue depend primarily on symptomatic and supportive care [2].

*Aconitum* plants contain diterpenoid alkaloids throughout the entire plant, with the highest concentrations found in the roots. To date, nearly a thousand diterpenoid alkaloids have been isolated from *Aconitum* plants. Their chemical structures are structurally diverse and can be divided into two groups according to their carbon skeletons, C19- and C20-diterpenoid alkaloids [20]. All C19-diterpenoid alkaloids sharing an identical skeleton can be subdivided into four subtypes according to the substituents present at C8 and C14: diester-diterpenoid alkaloids (DDAs) with an acetyl group at C8, monoester-diterpenoid alkaloids (MDAs) with a hydroxyl group at C8, amine-diterpenoid alkaloids (ADAs) with two hydroxyl groups at C8 and C14, and other C19-diterpenoid alkaloids [20]. These alkaloids exhibit remarkably complex and structurally diverse architectures, accompanied by a broad spectrum of chemical properties and biological activities, thereby constituting the fundamental chemical basis underlying both the medicinal efficacy and toxicity of *Aconitum* plants [20]. The pharmacological activities of some *Aconitum* alkaloids have been extensively investigated [21,22], revealing a complex profile of both therapeutic potential and toxicity. Among the alkaloids in *Aconitum* plants, DDAs exhibit the highest toxicity due to the two ester groups in their molecular structure, which is significantly higher than that of MDAs, ADAs, and C20-diterpenoid alkaloids, and they are often the most abundant alkaloids in certain *Aconitum* species [23,24]. Therefore, DDAs are the chemical components that require priority control and detection in both the medicinal use and poisoning incidents of Aconitum [23,24]. Aconitine and related diterpenoid alkaloids exhibit potent cardiotoxicity and neurotoxicity, as these compounds exert their toxic effects primarily through persistent activation of voltage-gated sodium channels, leading to life-threatening arrhythmias and respiratory paralysis [25]. Thus, the narrow therapeutic index of these alkaloids necessitates rigorous processing protocols, including prolonged decoction or alkaline hydrolysis, to reduce toxicity while preserving bioactivity [21].

The detection of *Aconitum* alkaloids plays a critical role in clinical toxicology, forensic investigations, and the determination of causative agents and trace-back analysis of poisoning incidents. Among all the alkaloids, aconitine is the primary target analyte for detection, followed by mesaconitine and hypaconitine, with targeted detection by LC-MS/MS being predominantly employed in poisoning analysis [26,27,28]. However, in certain poisoning cases, aconitine and its related alkaloids are not the causative agents. For instance, yunaconitine and its associated alkaloids were detected for the first time in the urine of poisoned patients [1], and jesaconitine was identified as the principal toxin responsible in a poisoning case in Japan [29]. These cases clearly demonstrate the limitations of relying solely on targeted detection of a limited number of toxins. Targeted detection methods are constrained by the limited availability of *Aconitum* alkaloids reference materials and certified reference standards, potentially leading to the failure to detect concealed or unknown toxins. The advantages of non-targeted screening based on liquid chromatography coupled to high resolution mass spectrometry, combined with molecular networking for the identification of structural analogues, offer a solution to this challenge.

In the present study, the *Aconitum* alkaloids in the tincture sample responsible for an *Aconitum* poisoning incident involving 15 victims with 5 fatalities were comprehensively characterized using non-targeted screening combined with molecular networking. The results demonstrated that pseudoaconitine, 8-deacetylpseudoaconitine, and 3′-methoxyacoforestinine were the causative agents of the poisoning. These alkaloids had not been previously reported in China, indicating that this poisoning was caused by previously undocumented alkaloids. These findings not only elucidate the causative agents of this specific incident and provide a methodological reference for *Aconitum* toxin detection, but also yield novel insights into the etiology of *Aconitum* poisoning.

## 2. Results

### 2.1. Aconitum Alkaloids Characterized in the Tincture

A total of 58 *Aconitum* alkaloids were identified from the tincture using LC-Q-TOF/MS (Table 1). Among them, 10-OH-neoline (**5**) was detected but below the limit of quantification, neoline (**11**, 9.18 mg/L), guan-fu base H (**15**, 82.83 mg/L), chasmanine (**18**, 14.40 mg/L), ludaconitine (**34**, 7.09 mg/L), 8-deacetylyunaconitine (**36**, 0.68 mg/L), falconeridine (**38**, 0.99 mg/L), indaconitine (**48**, 5.70 mg/L), and yunaconitine (**49**, 1.57 mg/L) were detected by comparison of their relative retention times, precursor ions, and MS/MS spectra with those of reference standards, and quantified. These results confirm that *Aconitum*-derived components are present in the tincture; nevertheless, these alkaloids are present only in trace amounts or exhibited lower toxicity and therefore should not be regarded as the principal cause of poisoning. Contrary to our expectations, the primary toxins typically associated with *Aconitum* poisoning, aconitine, mesaconitine, and hypaconitine, were notably absent (Figure S2; Supplementary Materials).

**Figure 1:**
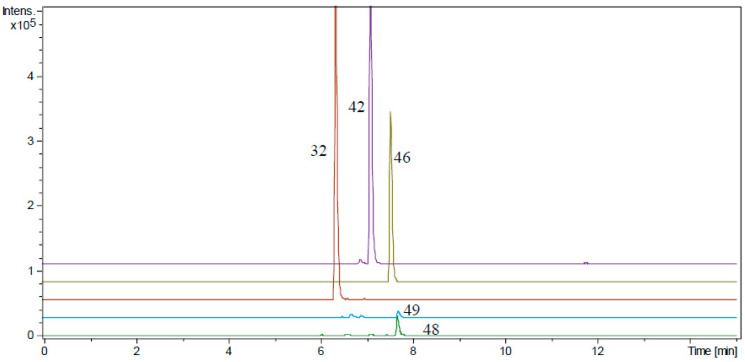
The extracted ion chromatograms of the three most abundant peaks (compounds **32**, **42**, and **46**), indaconitine (**48**) and yunaconitine (**49**).

Notably, three highly abundant unknown compounds (compounds **32**, **42**, and **46**) were observed in the mass spectrometric data. Their peak areas were 50.1-, 46.52-, and 27.21-fold higher than that of yunaconitine, and 17.05-, 15.83-, and 9.26-fold higher than that of indaconitine, respectively (Figure 1). These results suggest that these unidentified substances, rather than the common alkaloids typically responsible for *Aconitum* poisoning, may be the primary toxic agents in this incident. Unfortunately, reference standards for these compounds were unavailable for confirmation. Given that the *Aconitum* alkaloids detected in the tincture likely derive from *Aconitum* species, these unknown compounds were subsequently characterized through molecular networking and structure elucidation.

### 2.2. GNPS Molecular Networking

**Figure 2:**
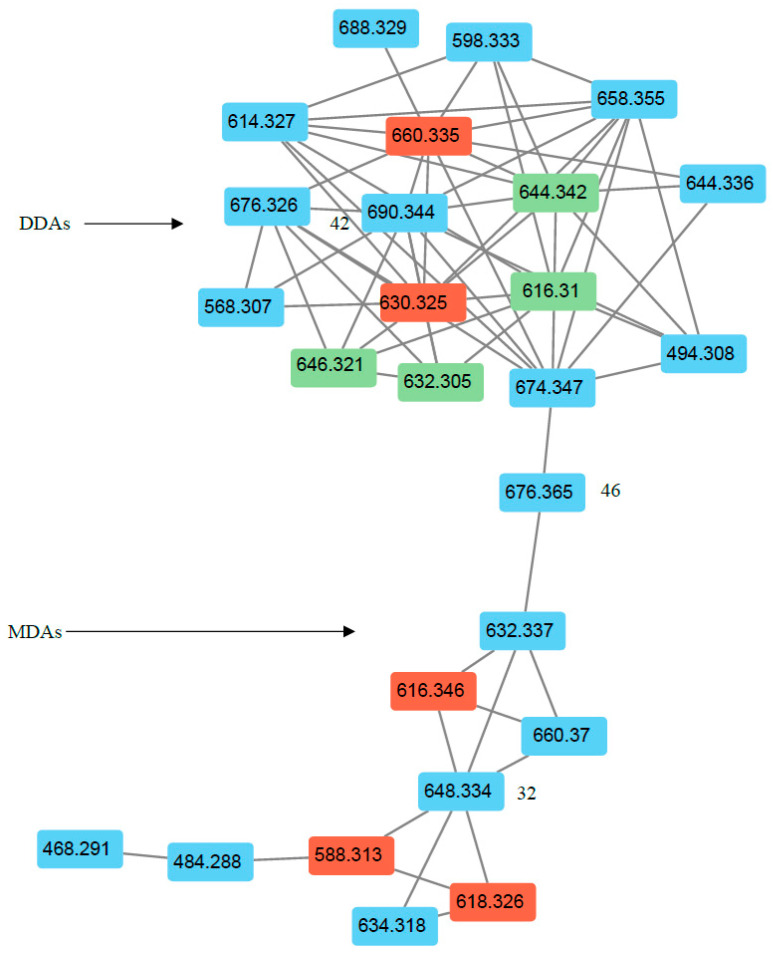
GNPS-based molecular network of tincture samples and reference standards (red: both in tincture and reference standards; green: in reference standards; blue: in tincture sample).

A molecular network was constructed using the *Aconitum* alkaloids detected in the tincture along with 17 reference alkaloids, resulting in a total of 26 nodes (Figure 2). Among these, 4 alkaloids were found exclusively in the reference standards, 5 were present in both the reference standards and the tincture sample, and 17 were detected only in the tincture. The molecular network consisted of two related clusters. One cluster comprised 16 nodes, including the DDAs aconitine, mesaconitine, hypaconitine, indaconitine, yunaconitine, and crassicauline A, suggesting that the compounds grouped in this cluster share similar structural features and should be classified as DDAs. The other cluster contained 10 nodes, including the MDAs ludaconitine, falconeridine, and 8-deacetylyunaconitine, indicating that the compounds in this cluster are likely MDAs. The three most abundant compounds in the tincture were all represented in the molecular network: compound **42** was classified as a DDA, whereas compounds **32** and **46** were classified as MDAs.

### 2.3. Identification of Diester-Diterpenoid Alkaloids

The MS characteristics and fragmentation patterns of DDAs have been extensively investigated [20,25,30,31,32,33,34]. According the literature, compounds meeting the following MS/MS criteria were classified as DDAs: (1) sequential neutral losses of acetic acid, methanol, water, and benzoic acid, 4-methoxybenzoic acid, or 3,4-dimethoxybenzoic acid in positive ion mode; (2) the base peak corresponding to the loss of the acetyl group under high collision energy; and (3) the presence of diagnostic fragment ions derived from the benzoyl (C_7_H_5_O^+^, *m*/*z*:105.3349), anisoyl (C_8_H_7_O_2_^+^, *m*/*z*:135.0440), vanilloyl (C_8_H_7_O_3_^+^, 151.0390) and veratroyl (C_9_H_9_O_3_^+^, *m*/*z*:165.0546) moiety.

A total of 15 DDAs were identified from the tincture. Among them, indaconitine and yunaconitine were identified by comparison with reference standards; 4 compounds were identified through PubChem database searching and structure assignment; and 2 were identified as isomers of pseudaconitine and crassicauline A, respectively (Figure S3; Supplementary Materials). The remaining seven DDAs could not be identified due to the lack of candidate structures.

**Figure 3:**
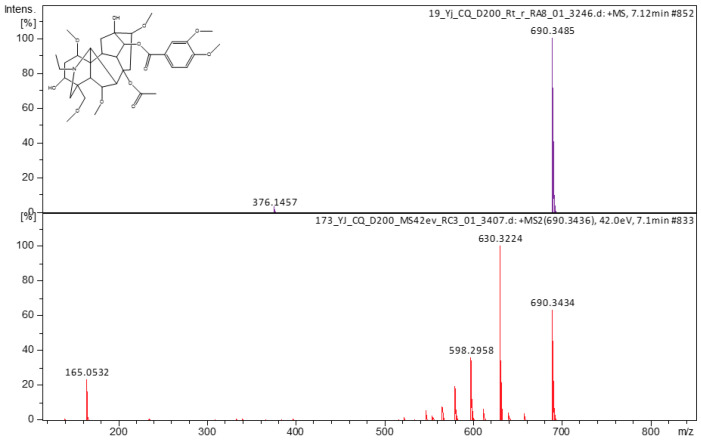
The MS, MS^2^ (CID, 42 eV), and the structure of pseudoaconitine.

Compound **42** was identified as pseudaconitine (Figure 3), with its structure and MS/MS spectrum being fully consistent. Structurally, it differs from yunaconitine in the C-14 ester substituent: pseudaconitine bears a 3,4-dimethoxybenzoate (veratroyl) group, whereas yunaconitine carries a 4-methoxybenzoate (anisoyl) group. This structural distinction was confirmed by the corresponding differences in their MS/MS profiles, specifically, pseudaconitine exhibited a diagnostic fragment ion at *m*/*z* 165.0546 (veratroyl, C_9_H_9_O_3_^+^), in contrast to yunaconitine’s characteristic fragmentation ion at *m*/*z* 135.0440 (anisoyl, C_8_H_7_O_2_^+^) and, thereby further validating the identification.

### 2.4. Identification of Monoester-Diterpenoid Alkaloids

MDAs differ structurally from DDAs by the absence of an acetyl group, typically at C-8. Consequently, the MS/MS fragmentation of MDAs is distinguished from that of DDAs by the lack of a cleavage pathway involving neutral loss of acetic acid (60 Da). Instead, MDAs exhibit fragmentation characterized by single ester neutral loss and the formation of diagnostic fragment ions containing the benzoyl, anisoyl, vanilloyl, and veratroyl moiety [25,34]. Compounds meeting these criteria were classified as MDAs.

A total of 17 MDAs were identified from the tincture. Among them, ludaconitine, 8-deacetylyunaconitine, and falconeridine were confirmed by comparison with reference standards (Table 1). Three compounds were assigned through PubChem database searching and structure elucidation (Figure S4; Supplementary Materials). However, ten compounds could not be identified due to the lack of candidate structures.

**Figure 4:**
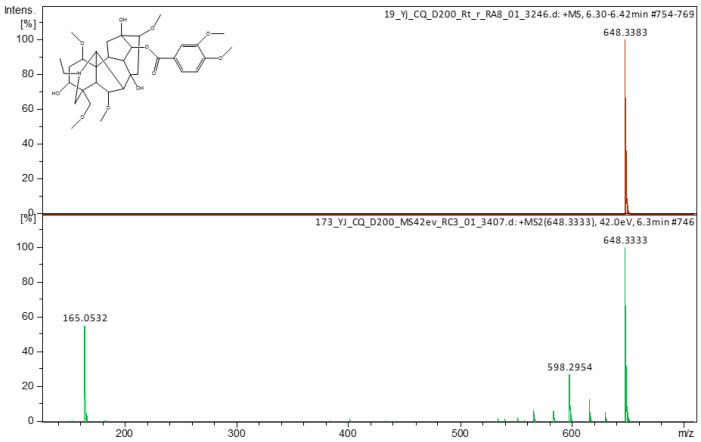
The MS, MS^2^ (CID, 42 eV), and structure of 8-deacetylpseudoaconitine.

Compound **32** was identified as 8-deacetylpseudaconitine (Figure 4), with its structure and MS/MS spectrum being fully consistent. Its structure is highly similar to those of both pseudaconitine and 8-deacetylyunaconitine. Compared with pseudaconitine, the structural distinction lies in the OH at C-8; this difference was accurately reflected in the MS/MS spectrum. In contrast to 8-deacetylyunaconitine, compound 32 bears a 3,4-dimethoxybenzoyl (veratroyl) group rather than a 4-methoxybenzoyl (anisoyl) group at C-14. This structural variation was unambiguously confirmed by the corresponding MS/MS spectral differences, specifically, the presence of the diagnostic *m*/*z* 165.0546 fragment ion (veratroyl, C_9_H_9_O_3_^+^), as opposed to the *m*/*z m*/*z* 135.0440 (anisoyl, C_8_H_7_O_2_^+^), thereby further validating the identification.

**Figure 5:**
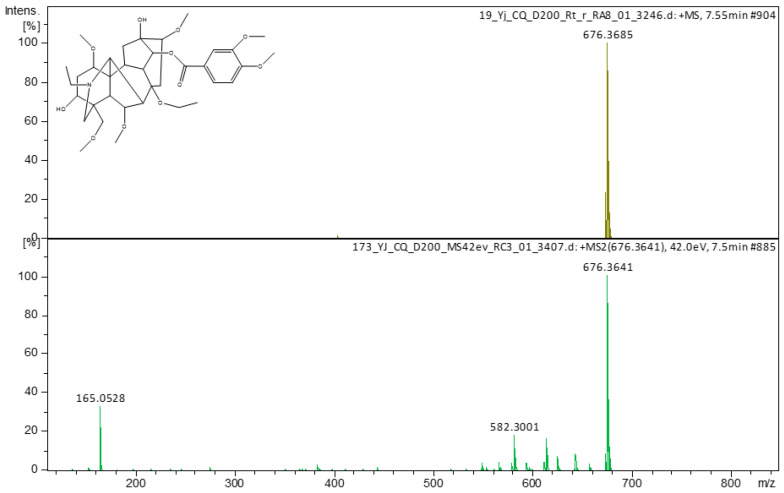
The MS, MS^2^ (CID, 42 eV), and the structure of 3′-methoxyacoforestinine.

Compound **46** was identified as 3′-methoxyacoforestinine (Figure 5). It differs from 8-deacetylpseudaconitine and pseudaconitine in the substituent at C-8: an ethoxy group replaces the hydroxyl group and acetyl, respectively. Consequently, compared with 8-deacetylpseudaconitine and pseudaconitine, its MS/MS spectrum exhibited a diagnostic neutral loss of ethanol (C_2_H_6_O, 46.0419 Da), a characteristic fragmentation pattern that further validated this structural assignment.

### 2.5. Identification of Amaine-Diterpenoid Alkaloids

Compared with DDAs and MDAs, ADAs lack ester groups in their structures. Their MS/MS fragmentation is therefore characterized by sequential losses of methanol and water, together with skeletal cleavage to yield fragment ions [35].

A total of 17 ADAs were identified from the tincture (Table 1). Among them, 10-OH-neoline, neoline, and chasmanine were confirmed by comparison with reference standards. An additional compound was assigned through PubChem database searching and structure elucidation (Figure S5; Supplementary Materials). Furthermore, only two compounds could be tentatively correlated with their MS/MS features and molecular structure. The remaining 12 ADAs were identified only at the class level, as their precise structures could not be determined.

### 2.6. Identification of C20-Diterpenoid Alkaloids

C20-diterpenoid alkaloids exhibit more complex and diverse structures. Guan-fu base H was detected in the tincture by comparison with a reference standard (Figure S6; Supplementary Materials). Compound **5** was tentatively identified as a C20-diterpenoid alkaloid, as its MS/MS spectrum showed only water loss (18.0105 Da), a fragmentation pattern consistent with the structural features of this class.

## 3. Discussion

On 3 May 2018, a poisoning incident occurred during a family dinner in Chongqing, China. The first case developed symptoms approximately one hour after the meal began, presenting with perioral numbness, chest tightness, nausea, and vomiting. Subsequently, 14 additional individuals experienced similar manifestations. One patient suffered sudden cardiac arrest and died in the ambulance on the way to the hospital. The remaining 14 patients were hospitalized with hypotension and various arrhythmias, including ventricular tachycardia, sinus tachycardia, atrioventricular block, and even ventricular flutter. Four of these patients died in the hospital from refractory arrhythmias despite aggressive resuscitation efforts [36]. Epidemiological investigation implicated the consumption of a self-prepared medicinal tincture as the cause of the incident; however, the botanical information and the source of the plant materials could not be determined because the tincture had been prepared several years earlier. To ascertain the cause of this poisoning event, toxicological analysis of the tincture was warranted.

Based on the clinical manifestations [2,13] of the patients (perioral numbness, chest tightness, nausea, vomiting, hypotension, and various arrhythmias) and the epidemiological characteristics of toxic plant poisoning in China [3,4], we preliminarily concluded that this incident was aconite poisoning. Previous studies [1,10,37,38] have confirmed that DDAs, including aconitine, mesaconitine, hypaconitine, and yunaconitine, are the primary causative agents of aconite poisoning. Therefore, we first applied our established targeted analytical method using the available *Aconitum* alkaloid reference standards in our laboratory to analyze the implicated tincture. Targeted analysis detected nine *Aconitum* alkaloids in the tincture, comprising two DDAs, three MDAs, three ADA, and one C20-diterpenoid alkaloid. These nine components all originated from *Aconitum* plants [25,39,40], confirming that the botanical materials in the tincture contained *Aconitum* species. Contrary to expectations, the most common DDAs and their related compounds known to be responsible for aconite poisoning were not detected. Meanwhile, the two detected DDAs, yunaconitine and indaconitine, were present at relatively low concentrations, which could not adequately explain the severity of the poisoning or the five deaths. Full scan MS data from ultra-high-performance liquid chromatography–quadrupole time-of-flight mass spectrometry (UHPLC-Q-TOF/MS) revealed three predominant compounds with peak areas substantially higher than those of yunaconitine and indaconitine, and indicated the presence of numerous unidentified chemicals. These compounds remained unidentified in the targeted analysis due to the lack of corresponding reference standards. We therefore inferred that other *Aconitum* toxins, not detected by the targeted methods, were present in the tincture, and that these constituents were likely the true causative agents of the severe poisonings and deaths. Therefore, the cause of this poisoning was still ambiguous and needed to be confirmed through further testing.

A notable characteristic of *Aconitum* plants is the coexistence of multiple structurally similar *Aconitum* alkaloids within the same species [20,34,41]. This structural similarity results in highly correlated mass spectrometric fragmentation behaviors, providing favorable conditions for the application of molecular networking based on MS/MS spectral similarity clustering. In recent years, molecular networking has been successfully employed for the identification of complex alkaloid constituents in *Aconitum* species [34]. Accordingly, the present study utilized the GNPS platform to construct molecular networks for both the implicated tincture and available reference standards, followed by comparison with the GNPS spectral library to uncover hidden alkaloids potentially present in the tincture. Under optimized GNPS parameters, 26 compounds were successfully clustered into two distinct classes: 12 DDAs and 7 MDAs. Particularly, the three most abundant unknown compounds in the tincture were all effectively incorporated into the molecular network, with one classified as a DDA and the other two as MDAs, strongly suggesting that these unknowns belong to the diester- or monoester-diterpenoid structural class and likely represent highly toxic constituents not covered by the targeted analytical method. However, three ADAs, namely 10-OH-neoline, neoline, and chasmanine, as well as one C20 diterpenoid alkaloid (guan-fu base H), failed to be included in the molecular network. Even after adjusting network construction parameters, such as reducing the minimum matched fragment ion number or relaxing the cosine similarity threshold, these compounds remained excluded in the molecular networking. Detailed analysis indicated that this exclusion primarily resulted from substantial differences in MS/MS fragmentation patterns: ADAs differ from DDAs/MDAs in their substitution patterns on the C19-diterpenoid skeleton, while C20 diterpenoid alkaloids possess fundamentally distinct skeletal architectures, thereby failing to meet the similarity matching criteria of molecular networking. Additionally, several low-abundance *Aconitum* alkaloids in the tincture could not be effectively clustered due to insufficient signal intensity or inadequate fragment ion information. The molecular networking results convincingly confirmed the presence of numerous DDAs and MDAs in the tincture that had escaped detection by targeted analysis; yet this approach was unable to provide specific structural information for these compounds. To achieve accurate identification of these concealed toxins, a non-targeted screening strategy employing high-resolution mass spectrometry was subsequently implemented for further structural elucidation.

GNPS database searching failed to match most compounds. First, based on accurate mass measurements of precursor ions, isotopic distribution patterns, and MS/MS fragment ion information from high-resolution mass spectrometry, molecular formulas were deduced for 58 compounds. According to characteristic fragmentation rules and diagnostic fragment ions specific to different classes of *Aconitum* alkaloids (Table 2), these compounds were preliminarily assigned to 15 DDAs, 17 MDAs,17 ADAs, and 2 C20-diterpenoid alkaloids. Among them, two DDAs, three MDAs, three ADAs, and one C20 alkaloid were accurately identified by comparison with available reference standards in our laboratory, while seven compounds remained unclassified due to insufficient characteristic fragments or inadequate evidence for classification. The deduced molecular formulas were then subjected to structure retrieval in the PubChem database to obtain candidate structures. Subsequently, fragment ions in the MS/MS spectra were matched against these candidate structures individually. Ultimately, reasonable structures showing high consistency between MS/MS spectral features and theoretical fragmentation patterns were obtained for 8 DDAs, 6 MDAs, and 3 ADAs. In contrast, 7 DDAs, 11 MDAs,12 ADAs, and 1 C20 alkaloid could not be reliably matched to any candidate structure, primarily for the following reasons: the MS/MS characteristics and diagnostic fragment ions were inconsistent with the fragmentation behavior of candidate structures; no candidate structures corresponding to the deduced molecular formulas were retrieved from the PubChem database. Fortunately, the three most abundant compounds in the tincture all successfully obtained candidate structures, with their MS/MS fragmentation patterns and diagnostic fragment ions being fully consistent with the proposed structures. These were identified as pseudoaconitine (DDA), 8-deacetylpseudoaconitine (MDA), and 3′-methoxyacoforestinine (MDA). To further validate these identifications, the MS/MS spectra and fragmentation behaviors of pseudoaconitine and 8-deacetylpseudoaconitine were comprehensively compared with those of structurally defined analogues, namely yunaconitine and 8-deacetylyunaconitine reference standards. Both pseudoaconitine-type compounds exhibited highly similar mass spectrometric fragmentation characteristics, diagnostic fragment ion distributions, and relative abundance patterns to their respective yunaconitine-type counterparts, thereby confirming the accuracy of their structural assignments. Additionally, the structure of 3′-methoxyacoforestinine was further confirmed through comparative MS/MS spectral analysis with 8-deacetylpseudoaconitine and pseudoaconitine, particularly through the observation of characteristic ethoxy related neutral loss fragments consistent with the proposed structural modification.

Compared with targeted analytical methods, the non-targeted screening strategy effectively overcame the limitations imposed by the availability of reference standards, enabling comprehensive characterization of compounds in the sample and thereby successfully revealing concealed toxins that were undetectable by conventional targeted approaches. Nevertheless, certain limitations of this method should be acknowledged. Although numerous studies have investigated the mass spectrometric characteristics and fragmentation mechanisms of *Aconitum* alkaloids, providing valuable information for structural identification [20,25,30,31,32,33,34], the majority of these studies have focused on a limited number of alkaloids or toxins derived from a restricted range of *Aconitum* species. Consequently, when confronted with alkaloids from novel or previously uncharacterized *Aconitum* species, the available reference information is insufficient, which largely explains why a considerable proportion of the alkaloids in this study could not be accurately identified. Furthermore, the completeness of existing public databases, such as PubChem and GNPS, constrains the candidate structure retrieval process, potentially resulting in failed matches for certain compounds. For structural analogues lacking characteristic diagnostic fragments or with unclear fragmentation mechanisms, accurate identification remains challenging.

To our knowledge, this was the first detection of pseudoaconitine, 8-deacetylpseudoaconitine, and 3′-methoxyacoforestinine in samples from a poisoning incident in China. Notably, these alkaloids have not been previously reported in phytochemical studies of Aconitum species distributed within China. Pseudoaconitine shares the same diester-diterpenoid structural skeleton as aconitine and the same mechanism of toxicity, but it is approximately 1.5 times more potent than aconitine [42]. Thus, pseudoaconitine was identified as the principal causative agent responsible for the onset of toxicity and the fatalities in this incident. Additionally, 8-deacetylpseudoaconitine and 3′-methoxyacoforestinine were present at high concentrations in the tincture and contributed substantially to the severity of the poisoning. Previous studies have reported that *Aconitum* species containing these alkaloids are predominantly distributed in South Asia, notably *Aconitum ferox* [42,43,44,45], and *Aconitum balfourii* [46]. However, systematic investigations into the taxonomic distribution and structural characteristics of these toxins remain lacking, which fundamentally accounts for the failure to accurately identify certain *Aconitum* alkaloids in the present study. We hypothesize that the *Aconitum* plants containing these toxins within China may be distributed in the border regions between Tibet and South Asia. Nevertheless, searches of major domestic herbarium collections failed to yield *Aconitum* specimens likely to contain these alkaloids; therefore, this hypothesis awaits further verification.

The findings of this study not only elucidate the definitive causative agents of this poisoning incident but also provide novel insights into the etiological spectrum of aconite poisoning. The analytical strategy and methodological framework established herein offer an important reference for the toxicant identification of future aconite poisoning events and have significant implications for forensic toxicology and public health emergency response (Appendix A, Table A1).

## 4. Conclusions

In this study, a stepwise strategy integrating targeted analysis, molecular networking, and untargeted screening was employed to systematically identify the toxic constituents in a medicinal tincture implicated in a mass poisoning incident that occurred in Chongqing, China, in May 2018, involving 15 victims and resulting in 5 fatalities. Epidemiological investigation and clinical manifestations suggested aconite poisoning resulting from accidental consumption of a tincture containing *Aconitum* ingredients. Targeted screening based on 17 *Aconitum* alkaloid reference standards using LC-MS identified nine alkaloids in the tincture, including two DDAs, indaconitine and yunaconitine, confirming the presence of *Aconitum*-derived botanical materials. However, these findings were insufficient to account for the severity of the poisoning. Notably, three predominant compounds remained unidentified by targeted screening, while the most common DDAs typically implicated in *Aconitum* poisoning, namely aconitine, mesaconitine, hypaconitine, and their related alkaloids, were not detected. These results indicated the presence of numerous uncharacterized *Aconitum* alkaloids in the tincture. Consequently, GNPS-based molecular networking was introduced, which successfully clustered the high-abundance unknown compounds into DDA and MDA classes, suggesting the existence of other highly toxic diester- or monoester-type alkaloids. Further integration with high-resolution mass spectrometry-based untargeted screening led to the identification of 58 *Aconitum* alkaloids in total. The three most abundant compounds were accurately identified as pseudoaconitine, 8-deacetylpseudoaconitine, and 3′-methoxyacoforestinine.

To our knowledge, this represents the first detection of pseudoaconitine-type alkaloids in poisoning incident samples in China. Pseudoaconitine, a highly toxic diester-diterpenoid alkaloid, was identified as the principal causative agent responsible for the poisonings and fatalities in this incident, while 8-deacetylpseudoaconitine and 3′-methoxyacoforestinine, both present at high concentrations, made substantial contributions to the severity of the poisoning. These findings not only elucidate the true etiology of this unusual *Aconitum* poisoning event but also demonstrate that the integrated analytical strategy effectively overcomes the limitations of conventional targeted methods constrained by reference standard availability. The methodology established herein provides a robust framework for comprehensive toxicant identification in complex matrices of unknown botanical origin and offers an important reference for the detection and investigation of future aconite poisoning incidents, with significant implications for clinical toxicology, forensic toxicology, and public health emergency response.

## 5. Materials and Methods

### 5.1. Chemicals and Regents

Reference standards of aconitine, mesaconitine, hypaconitine, yunaconitine, crassicauline A, indaconitine, 8-deacetylyunaconitine, songorine, delsoline, and neoline (all with HPLC purity > 98%) were purchased from Chengdu Must Bio-Technology Co., Ltd. (Chengdu, China). Reference standards of 14-Benzoyl-8-O-methylaconine, falconeridine, and 10-OH-neoline (all with HPLC purity > 97%) were provided by BioBioPha (Kunming, China). Reference standard of 12-epinapelline, guan-fu base H, andludaconitine(HPLC purity > 98%) was purchased from Chengdu Biopurify Phytochemicals Ltd. (Chengdu, China). 6-benzoylheteratisine (purity ≥ 98%) was purchased from Chengdu Alfa Biotechnology Co., Ltd. (Chengdu, China). LC-MS grade formic acid, acetonitrile, water, and isopropanol were obtained from Thermo Fisher Scientific Inc. (Waltham, MA, USA). Analytical reagent (AR) grade sodium hydroxide was obtained from Sinopharm Chemical Reagent Co., Ltd. (Beijing, China). A 10 mM sodium formate calibration solution was prepared by diluting 1 mol/L aqueous sodium hydroxide solution with isopropanol-water (1:1, *v*/*v*) containing 0.2% formic acid.

### 5.2. Reference Standard Solutions Preparation and Sample Pretreatment

All toxin reference standards were accurately weighed (5.0 mg each) into individual volumetric flasks and dissolved in acetonitrile to a final volume of 5 mL, yielding stock solutions at a concentration of 1.0 mg/mL, which were stored at −80 °C. A mixed intermediate solution was prepared by diluting the stock solutions to 10 μg/mL with acetonitrile. The working solution was prepared by diluting the mixed intermediate solution with the initial mobile phase consisting of acetonitrile–water (15:85, *v*/*v*) containing 0.1% formic acid.

The tincture sample was stored at −80 °C prior to analysis. The tincture was diluted 200-fold with the initial mobile phase consisting of acetonitrile–water (15:85, *v*/*v*) containing 0.1% formic acid, centrifuged at 15,000 rpm for 10 min, and the supernatant was transferred to 2 mL autosampler vials for analysis.

### 5.3. LC-Q-TOF/MS Analysis

The LC-Q-TOF/MS analysis was performed using an Ultimate 3000 UHPLC system (Thermo Fisher Scientific, Germering, Germany) coupled to a microTOF-Q III mass spectrometer (Bruker Daltonics, Bremen, Germany). The mass spectrometer was equipped with an electrospray ionization (ESI) source operated in positive ion mode with the following parameters: capillary voltage, 4.5 kV; nebulizer gas, nitrogen at 1.5 bar; dry gas, nitrogen at 8 L/min; dry temperature, 250 °C. The data acquisition rate was set at 2 Hz. Quantitative analysis was performed using full-scan MS mode. Screening and confirmation were performed using MS/MS experiments at collision energies of 36.0 and 42.0 eV (CID), respectively.

An ACQUITY UPLC BEH C18 column (2.1 × 150 mm, 1.7 μm; Waters, Milford, MA, USA) was employed as the stationary phase for chromatographic separation. The mobile phase consisted of 0.1% formic acid in water (A) and 0.1% formic acid in acetonitrile (B). The flow rate was 0.3 mL/min. The gradient program was as follows: 15% B held for 0.5 min, increased to 20% B at 2.5 min, 35% B at 3.5 min, 40% B at 4.0 min, 65% B at 8.5 min, 90% B at 9.0 min, 95% B at 10.5 min, held at 95% B for 3.0 min, and then returned to the initial conditions over 1.5 min for column re-equilibration. The total run time was 15.0 min, with a 4.0 min re-equilibration period between injections. The column temperature was maintained at 40 °C, and the injection volume was 5 μL. Prior to each sample analysis, 20 μL of 10 mM sodium formate calibration solution was injected via a six-port valve switch at 0.01–0.5 min for real-time mass calibration of the instrument. 

### 5.4. Data Analysis

The acquired MS and data-dependent MS/MS (Auto MS/MS) spectrometric data were processed using DataAnalysis 4.4 software (Bruker Daltonics, Bremen, Germany) for mass calibration and molecular formula calculation. The accurate mass accuracy of all sodium formate calibration clusters was within 2 ppm. Compound molecular formula identification followed these criteria: accurate mass error < 5 ppm (<10 ppm for lower abundance compounds), relative retention time deviation < 0.01 min, and isotopic pattern mSigma value < 50. The deduced molecular formula was subjected to structure retrieval in the PubChem database (https://pubchem.ncbi.nlm.nih.gov/, accessed on 20 March 2026) to obtain candidate structures. The structures were elucidated based on fragmentation rules and reference standards using MS/MS.

### 5.5. Global Natural Products Social Molecular Networking

The raw data-dependent MS/MS (Auto MS/MS) spectrometric data were calibrated and subsequently converted to mzXML format by DataAnalysis 4.4 (Bruker Daltonics, Bremen, Germany). The converted data were then uploaded to the GNPS platform (https://gnps.ucsd.edu/ProteoSAFe/static/gnps-splash.jsp, accessed on 10 February 2026) for molecular networking. The parameters for molecular network construction were set as follows: precursor ion mass tolerance, 0.01 Da; fragment ion mass tolerance, 0.01 Da; minimum pairs cosine, 0.7; and minimum cluster size, 3. The minimum number of matched fragment ions was optimized among 6, 5, 4, and 3. The molecular network was visualized using Cytoscape 3.10.4 [47].

## Figures and Tables

**Table 1:** Information on 58 *Aconitum* alkaloids identified or characterized in the poisoning-implicated tincture.

Compound	RT	Molecular Formula	*m*/*z* Calculated [M+H]^+^	*m*/*z* Measured [M+H]^+^	Mass Error [mDa]	Mass Error [ppm]	mSigma	MS^2^ Fragmentation Ions ^#^ (Relative Abundance, %)	Class	Identification
**1**	1.52	C_23_H_35_NO_6_	422.2537	422.2511	2.7	6.3	30.0	308.2000 (23.0), 344.2248 (61.1), 404.2386 (100.0)	ADA	
**2**	1.70	C_23_H_37_NO_6_	424.2694	424.2683	1.1	2.5	16.7	338.2115 (16.4), 356.2312 (31.8), 388.2402 (67.4), 406.2558 (91.8), 424.2656 (100.0)	ADA	
**3**	1.97	C23H37NO5	408.2744	408.2728	1.6	3.9	24.5	154.1202 (31.6), 358.2425 (28.3), 390.2677 (100.0), 408.2777 (16.6)	ADA	
**4**	2.03	C_23_H_37_NO_6_	424.2694	424.2689	0.4	1.0	17.3	251.1400 (15.7), 388.2633 (23.0), 406.2553 (42.6), 424.2713 (100.0)	ADA	
**5**	2.05	C_24_H_39_NO_7_	454.2799	454.2785	1.5	3.2	36.9	404.2409 (40.5), 436.2703 (100.0), 454.2762 (45.4)	ADA	10-OH-neoline *
**6**	2.12	C_22_H_33_NO_3_	360.2533	360.2523	1.0	2.8	8.4	342.2317 (17.6), 360.2538 (100.0)	C20	
**7**	2.16	C_25_H_41_NO_8_	484.2905	484.2893	1.1	2.4	5.2	370.1970 (11.0), 402.2277 (17.6), 420.2447 (17.1), 434.2545 (54.0), 452.2651 (28.9), 484.2933 (100.0)	ADA	pseudoaconine
**8**	2.66	C_22_H_33_NO_3_	360.2533	360.2522	1.1	3.0	32.6	342.2425 (100.0)	unidentified	
**9**	2.78	C_23_H_33_NO_5_	404.2431	404.2418	1.4	3.4	5.5	326.2075 (83.3), 386.2303 (100.0)	unidentified	
**10**	3.22	C_20_H_23_NO_4_	342.1700	342.1680	2.0	5.7	10.6	191.0810 (39.8), 222.0690 (100.0)	unidentified	
**11**	3.24	C_24_H_39_NO_6_	438.2850	438.2840	1.0	2.3	4.6	154.1223 (45.7), 324.2015 (11.8), 356.2194 (37.4), 362.2324 (19.9), 370.2427 (15.4), 388.2467 (75.4), 420.2740 (100.0), 438.2827 (96.0)	ADA	neoline *
**12**	3.29	C_25_H_41_NO_7_	468.2956	468.2938	1.8	3.8	4.1	386.2306 (17.5), 404.2382 (23.8), 418.2536 (43.4), 436.2644 (19.5), 450.2806 (14.8), 468.2972 (100.0)	ADA	
**13**	3.30	C_27_H_43_NO_9_	526.3011	526.2995	1.6	3.0	6.1	430.2281 (15.1), 444.2367 (21.0), 462.2390 (16.8), 476.2647 (56.7), 494.2726 (35.1), 508.2819 (13.0), 526.2955 (100.0)	ADA	
**14**	4.11	C_24_H_39_NO_5_	422.2901	422.2864	3.7	8.8	25.9	108.0771 (40.2), 390.2591 (100.0), 422.2934 (56.1)	ADA	talatisamine
**15**	4.41	C_22_H_33_NO_2_	344.2584	344.2577	0.7	2.0	5.1	326.2470 (16.0), 344.2587 (100.0)	C20	guan-fu base H *
**16**	4.92	C_26_H_41_NO_8_	496.2905	496.2893	1.2	2.4	7.0	108.0854 (48.2), 122.0991 (24.6), 152.1066 (30.0), 154.1233 (39.7), 386.2342 (26.4), 414.2290 (46.2), 418.2641(18.2), 446.2521 (100.0), 478.2921 (26.4), 496.2828 (100.0)	ADA	
**17**	4.92	C_26_H_41_NO_7_	480.2956	480.2953	0.3	0.6	5.8	108.0825(66.2), 221.1297 (40.3), 430.2570 (41.2), 462.2850 (100.0), 480.2967 (60.6)	ADA	
**18**	5.12	C_25_H_41_NO_6_	452.3007	452.2993	1.4	3.0	4.5	356.2226 (30.1), 362.2283 (15.9), 388.2501 (45.2), 420.2730 (100.0), 452.3049 (85.3)	ADA	chasmanine *
**19**	5.26	C_23_H_31_NO_5_	402.2275	402.2262	1.3	3.2	10.4	342.2068 (93.8), 402.2251 (100.0)	MDA	
**20**	5.47	C_27_H_45_NO_7_	496.3269	496.3242	2.7	5.4	31.2	386.2273 (38.3), 464.2846 (24.1), 496.3320 (100.0)	ADA	
**21**	5.60	C_27_H_43_NO_7_	494.3112	494.3097	1.5	3.1	30.1	338.2109 (6.0), 370.2355 (27.1), 374.2363 (8.8), 402.2637 (100.0), 434.2924 (58.7), 494.3117 (7.4)	DDA	
**22**	5.75	C_29_H_45_NO_10_	568.3116	568.3099	1.8	3.1	16.6	458.2567 (36.1), 476.2630 (52.8), 508.2893 (100.0), 568.2994 (34.9)	MDA	
**23**	5.79	C_23_H_31_NO_3_	370.2377	370.2346	3.1	8.3	32.2	310.2162 (100.0), 370.2386 (35.6)	unidentified	
**24**	5.87	C_27_H_43_NO_8_	510.3061	510.3045	1.6	3.1	9.7	108.0775 (100.0), 154.1272 (28.2), 414.2214 (44.6), 446.2554 (76.5), 510.3019 (82.1)	ADA	
**25**	5.88	C_27_H_43_NO_7_	494.3112	494.3087	2.5	5.1	7.6	154.1230 (60.5), 398.2394 (100.0), 430.2715 (53.2), 462.2858 (79.0), 494.2976 (96.0)	MDA	
**26**	5.89	C_33_H_47_NO_11_	634.3222	634.3210	1.2	1.9	9.5	151.0376 (41.3), 552.2523 (7.6), 570.2660 (7.7), 584.2864 (35.5), 602.2953 (14.2), 616.3028 (7.4), 634.3205 (100.0)	MDA	
**27**	6.02	C_27_H_45_NO_6_	480.3320	480.3306	1.3	2.8	4.9	370.2368 (29.9), 384.2532 (27.7), 402.2691 (14.6), 416.2844 (24.7), 448.3044 (25.1), 480.3340 (100.0)	ADA	
**28**	6.08	C_40_H_53_NO_16_	804.3437	804.3409	2.8	3.5	17.6	598.2940 (18.5), 630.3241 (100.0), 804.3360 (49.1)	unidentified	
**29**	6.21	C_29_H_47_NO_9_	554.3324	554.3312	1.1	2.1	12.2	154.1278 (15.8), 432.2391 (15.6), 504.2848 (15.6), 554.3293 (100.0)	ADA	
**30**	6.30	C_33_H_47_NO_10_	618.3273	618.3264	0.9	1.4	31.4	151.0371 (42.7), 568.2951 (24.4), 586.2882 (20.4), 590.6207 (13.3), 618.3261 (100.0)	MDA	
**31**	6.32	C_38_H_55_NO_13_	734.3746	734.3720	2.6	3.5	17.1	630.3203 (93.8), 734.3670 (100.0)	unidentified	
**32**	6.37	C_34_H_49_NO_11_	648.3378	648.3382	−0.4	−0.6	14.0	165.0548 (57.3), 566.2764 (6.5), 584.2834 (6.9), 598.3022 (27.4), 616.3122 (13.5), 630.3258 (5.6), 648.3381 (100.0)	MDA	8-deacetylpseudoaconitine
**33**	6.45	C_35_H_49_NO_12_	676.3328	676.3309	1.8	2.7	12.9	151.0384 (20.0), 566.2729 (22.6), 584.2866 (36.5), 586.2930 (5.2), 616.3116 (100.0), 676.3280 (44.4).	DDA	
**34**	6.64	C_32_H_45_NO_9_	588.3167	588.3152	1.5	2.6	1.4	105.0345 (23.1), 524.2686 (9.8), 538.2772 (48.7), 556.2865 (19.6), 570.3128 (8.3), 588.3141 (100.0)	MDA	ludaconitine *
**35**	6.70	C_35_H_49_NO_11_	660.3378	660.3354	2.5	3.8	50.0	128.0739 (15.3), 165.0602 (43.8), 536.2577 (23.1), 568.2893 (92.0), 600.3173 (100.0), 660.3276 (52.5)	DDA	
**36**	6.75	C_33_H_47_NO_10_	618.3273	618.3255	1.8	2.9	9.5	135.0450 (91.5), 536.2738 (11.1), 554.2822 (11.0), 568.2844 (45.1), 586.2958 (15.5), 600.3298 (6.4), 618.3257 (100.0)	MDA	8-deacetylyunaconitine *
**37**	6.80	C_35_H_51_NO_11_	662.3535	662.3515	2.0	3.0	11.0	151.0374 (35.5), 554.2713 (14.4), 612.3236 (19.9), 662.3511 (100.0)	MDA	
**38**	6.85	C_34_H_49_NO_10_	632.3429	632.3418	1.2	1.9	2.9	165.0552 (83.7), 550.2743 (5.3), 568.2911 (9.2), 582.3081 (18.9), 600.3141 (31.4), 632.3414 (100.0)	DDA	
**39**	6.90	C_36_H_51_NO_12_	690.3484	690.3463	2.1	3.1	10.9	165.0550 (14.3), 598.3000 (13.2), 612.3166 (5.9), 630.3276 (100.0), 690.3500 (32.8)	DDA	pseudoaconitine isomer
**40**	6.93	C_35_H_49_NO_11_	660.3378	660.3365	1.4	2.1	50.3	151.0394 (18.3), 568.2856 (32.3), 600.3217 (100.0), 660.3306 (59.4)	DDA	
**41**	7.08	C_33_H_45_NO_9_	600.3167	600.3139	2.8	4.7	44.1	105.0341 (12.5), 476.2531 (28.8), 508.2684 (83.3), 540.2942 (100.0)	DDA	delphinine
**42**	7.12	C_36_H_51_NO_12_	690.3484	690.3483	0.1	0.2	11.5	165.0546 (24.8), 566.2736 (8.3), 580.2900 (18.2), 598.3020 (35.3), 630.3276 (100.0), 690.3496 (66.9)	DDA	pseudoaconitine
**43**	7.14	C_32_H_45_NO_7_	556.3269	556.3233	3.6	6.5	18.3	340.2394 (34.9), 372.2527 (100.0), 404.2798 (59.7)	MDA	
**44**	7.36	C_29_H_33_NO_5_	476.2431	476.2423	0.8	1.7	16.7	105.0350 (6.6), 294.1854 (17.6), 354.2083 (100.0), 356.2076 (5.2), 416.2130 (25.2), 476.2440 (90.5)	MDA	
**45**	7.42	C_34_H_49_NO_9_	616.3480	616.3475	0.6	0.9	20.3	154.1197 (13.3), 165.0550 (93.6), 552.2883 (31.2), 584.3236 (24.3), 616.3449 (100.0)	MDA	falconeridine *
**46**	7.56	C_36_H_53_NO_11_	676.3691	676.3685	0.6	0.9	4.2	165.0548 (36.6), 582.3068 (13.3), 612.3134 (5.0), 626.3309 (6.6), 644.3428 (8.4), 676.3709 (100.0)	MDA	3′-methoxyacoforestinine
**47**	7.57	C_36_H_51_NO_11_	674.3535	674.3525	1.0	1.5	51.8	165.0551 (49.1), 538.292 (5.2), 550.2771 (11.9), 564.2950 (18.4), 582.3078 (47.0), 584.3069 (5.0), 596.3238 (5.8), 614.3327 (100), 616.3457 (10.9), 674.3562 (68.4)	DDA	bikhaconitine
**48**	7.71	C_34_H_47_NO_10_	630.3273	630.3252	2.0	3.2	2.6	105.0315 (7.7), 506.2521 (12.2), 520.2746 (23.1), 538.2768 (46.7), 552.2952 (5.3), 570.3067 (100.0), 630.3242 (36.0)	DDA	indaconitine *
**49**	7.72	C_35_H_49_NO_11_	660.3378	660.3354	2.5	3.7	6.5	135.0426 (28.9), 550.2930 (20.7), 568.2906 (36.7), 582.3009 (6.2), 600.3159 (100.0), 660.3341 (45.6)	DDA	yunaconitine *
**50**	7.91	C_36_H_49_NO_12_	688.3328	688.3318	0.9	1.3	20.8	135.0419 (21.2), 550.2828 (30.8), 596.2795 (12.0), 628.3111 (100.0), 688.3443 (47.3)	DDA	
**51**	8.03	C_38_H_55_NO_12_	718.3797	718.3774	2.3	3.2	10.6	630.3278 (100.0)	unidentified	
**52**	8.09	C_36_H_53_NO_10_	660.3742	660.3732	1.0	1.6	5.2	165.0541 (38.0), 550.2829 (5.9), 564.3097 (5.2), 596.3280 (5.2), 610.3296 (8.6), 628.3495 (13.0), 660.3701 (100.0)	MDA	
**53**	8.14	C_34_H_47_NO_9_	614.3324	614.3324	0.0	0.0	46.0	105.0306 (12.9), 286.1242 (6.9), 444.2088 (6.2), 490.2505 (32.1), 522.2768 (100.0), 524.2899 (12.3), 554.3129 (70.7), 614.3437 (27.9)	DDA	chasmaconitine
**54**	8.20	C_36_H_51_NO_10_	658.3586	658.3568	1.8	2.7	50.7	165.0532 (42.8), 534.2805 (20.7), 566.3116 (100.0), 598.3406 (91.7), 626.3334 (7.0), 658.3565 (59.2)	DDA	
**55**	8.21	C_35_H_51_NO_10_	646.3586	646.3561	2.4	3.8	18.9	135.0452 (30.2), 596.3248 (31.7), 646.3556 (100.0)	MDA	acoforestinine(8-O-etylyunaconitine)
**56**	8.29	C_34_H_49_NO_9_	616.3480	616.3475	0.5	0.9	11.5	105.0327 (13.2), 506.2505 (6.8), 534.2818 (8.2), 552.2949 (6.7), 566.3107 (11.4), 584.3214 (14.6), 616.3493 (100.0)	MDA	
**57**	8.34	C_35_H_49_NO_10_	644.3429	644.3405	2.4	3.7	49.2	135.0400 (28.0), 552.2865 (35.1), 584.3190 (100.0), 586.3278 (13.6), 644.3398 (90.3)	DDA	crassicauline A isomer
**58**	8.85	C_34_H_47_NO_8_	598.3374	598.3349	2.5	4.2	46.6	105.0347 (5.6), 474.2653 (16.3), 506.2857 (100.0), 538.3135 (49.0), 598.3409 (34.4)	DDA	

Note: DDA, diester-diterpenoid alkaloids; MDA, monoester-diterpenoid alkaloids; ADA, amine-diterpenoid alkaloids; C20, C20-diterpenoid alkaloids. Unidentified: the compound could not be classified based on MS data. * Compounds identified by structural comparison with reference standards. ^#^ Fragment ions with relative abundances below 5.0% were removed.

**Table 2:** Structural characteristics and diagnostic fragments of different classes of *Aconitum* alkaloids.

Class	Structural Characteristics	Key Diagnostic Fragment
DDAs	The skeleton contains both one acetyl group and one aromatic acyl group, or alternatively, two acetyl groups.	Acetyl group: neutral loss of acetic acid, [M+H]^+^–CH_3_COOH. Aromatic acyl group: diagnostic fragment ions derived from the benzoyl (C_7_H_5_O^+^, *m*/*z* 105.0335), anisoyl (C_8_H_7_O_2_^+^, *m*/*z* 135.0440), vanilloyl (C_8_H_7_O_3_^+^, *m*/*z* 151.0390), and veratroyl (C_9_H_9_O_3_^+^, *m*/*z* 165.0546) moieties; or neutral loss of the corresponding acyl groups: benzoyl (–C_7_H_6_O_2_), anisoyl (–C_8_H_8_O_3_), vanilloyl (–C_8_H_8_O_4_), and veratroyl (–C_9_H_10_O_4_). Two acetyl groups: sequential neutral loss of two acetic acid molecules, [M+H]^+^–CH_3_COOH and [M+H]^+^–2CH_3_COOH.
MDAs	The skeleton contains only one acetyl group or one aromatic acyl group.	The characteristic diagnostic ions arising from the ester groups are identical to those of DDAs; however, only the diagnostic fragment ions corresponding to a single acetyl or aromatic acyl group are present.
ADAs	The skeleton lacks ester groups and is primarily substituted with hydroxyl and methoxy groups.	The characteristic ions derived from ester groups were absent. Instead, fragment ions were predominantly observed as a result of sequential neutral losses of water ([M+H]^+^–H_2_O) and methanol ([M+H]^+^–CH_3_OH).
C20-Diterpenoid Alkaloids	The skeleton is primarily substituted with hydroxyl groups, and exhibits considerable structural diversity.	Neutral loss of water yields [M+H]^+^–H_2_O; however, other structural factors must also be taken into consideration.

## Data Availability

The original contributions presented in this study are included in the article/Supplementary Material. Further inquiries can be directed to the first author.

## Supplementary Materials

The following supporting information can be downloaded at: https://www.mdpi.com/article/10.3390/toxins18060255/s1, Figure S1: The total ion chromatogram of the tincture sample using LC-Q-TOF/MS in full scan mode; Figure S2: The extracted ion chromatogram of aconitine (a), mesaconitine (b), and hypaconitine (c) in the tincture sample; Figure S3: The structures of DDAs identified in the tincture; Figure S4: The structures of MDAs identified in the tincture; Figure S5: The structures of ADAs identified in the tincture; Figure S6: The structure of C20-diterpenoid alkaloids identified in the tincture; Table S1: Retention times of *Aconitum* alkaloids in the tincture on an HSS T3 column.

## Abbreviations

The following abbreviations are used in this manuscript:

GNPS	Global Natural Products Social Molecular Networking
DDA	Diester-diterpenoid alkaloids
MDA	Monoester-diterpenoid alkaloids
ADA	Amine-diterpenoid alkaloids
LC-Q-TOF/MS	Liquid chromatography quadrupole time-of-flight Mass Spectrometry

## Appendix A

The integrated strategy developed herein is readily transferable to other laboratories, such as clinical toxicology and forensic laboratories. The entire workflow requires only LC-HRMS instrumentation and freely accessible web-platform (GNPS), with no dependency on proprietary platforms or rare reference standards. For laboratories lacking specific alkaloid standards, tentative identification can be achieved through diagnostic fragmentation ion and molecular networking classification alone, thereby enabling rapid screening of unknown *Aconitum*-containing samples.

**Table A1:** Key Steps of the Integrated Analytical Strategy.

Step	Procedure	Purpose
1	Targeted screening: LC-MS/MS analysis employing acquired reference standards.	Detect known *Aconitum* alkaloids; confirm presence of *Aconitum* ingredients.
2	Full MS scan with high-resolution mass spectrometry.	Discover compounds that targeted analysis may overlooked.
3	Data-dependent acquisition (DDA): Full-scan MS with automatic MS/MS triggering.	Generate MS/MS spectra for molecular networking and untargeted identification.
4	Molecular networking (GNPS): Cluster MS/MS spectra by spectral similarity.	Classify unknown compounds into DDA/MDA/ADA categories; identify hidden toxins.
5	Untargeted screening: Feature detection, formula prediction, database search.	Identify novel or uncharacterized alkaloids without reference standards, and obtain the candidate structure.
6	Structural confirmation: Compare diagnostic ions and mass spectral characteristics against candidate structures.	Verify alkaloids class and substitution pattern using rules in Table 2, Compare with the structure and mass spectra of structurally characterized compounds.
