# Supplementary material for: Unveiling Hidden Aconitum Alkaloids in a Poisoning-Implicated Tincture by Untargeted Screening and Molecular Networking
Source: Toxins (Basel). 2026 Jun 5;18(6):255. doi: 10.3390/toxins18060255 (PMC13308474; doi:10.3390/toxins18060255)
Supplement: Supplementary file 1 [file toxins-18-00255-s001.zip › toxins-4352396-supplementary.pdf]

Supplementary Materials: Unveiling Hidden *Aconitum* Alkaloids in a Poisoning-Implicated Tincture by Untargeted Screening and Molecular Networking

Qian He, Micong Jin, Jing Zhou, Hongshun Zhang and Chengye Sun

Contents

Figure S1. The total ion chromatogram of the tincture sample using LC-Q-TOF/MS in full scan mode.

Figure S2. The extracted ion chromatogram of aconitine (a), mesaconitine (b), and hypaconitine (c) in the tincture sample.

Figure S3. The structures of DDAs identified in the tincture.

Figure S4. The structures of MDAs identified in the tincture.

Figure S5. The structures of ADAs identified in the tincture.

Figure S6. The structure of C20-diterpenoid alkaloids identified in the tincture.

Table S1. Retention times of *Aconitum* alkaloids in the tincture on an HSS T3 column.

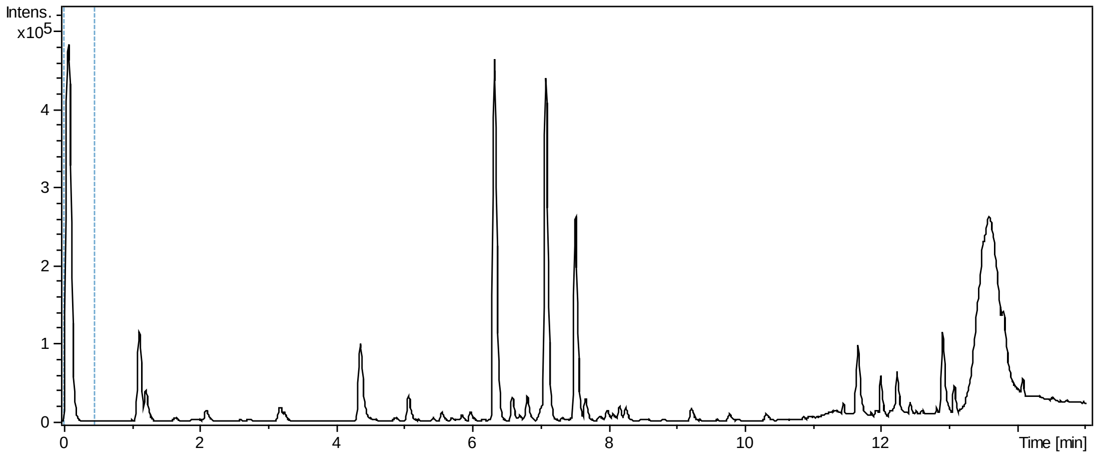

Figure S1. The total ion chromatogram of the tincture sample using LC-Q-TOF/MS in full scan mode

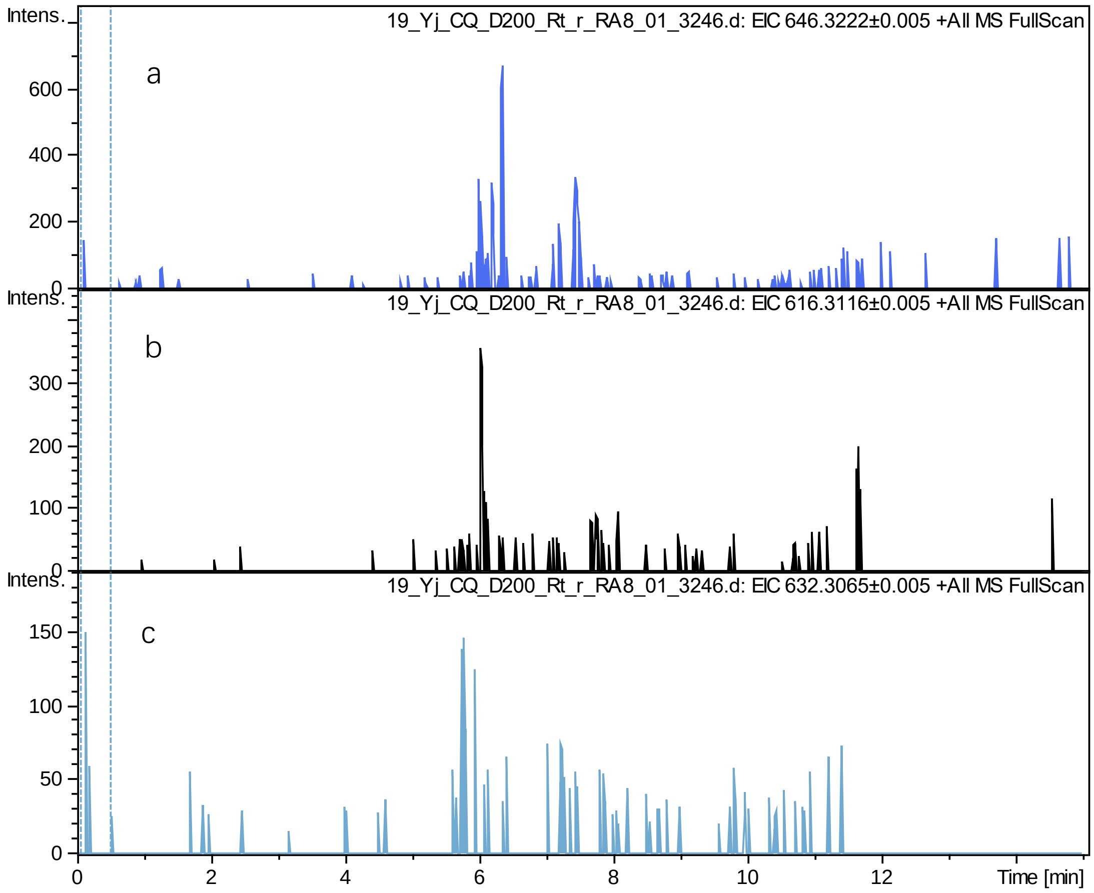

Figure S2. The extracted ion chromatogram of aconitine (a), mesaconitine (b), and hypaconitine (c) in the tincture sample.

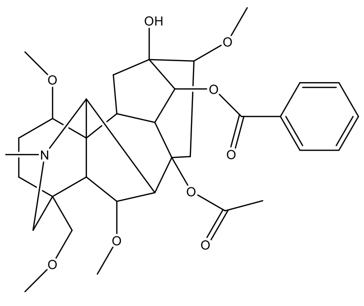

Delphinine (41)

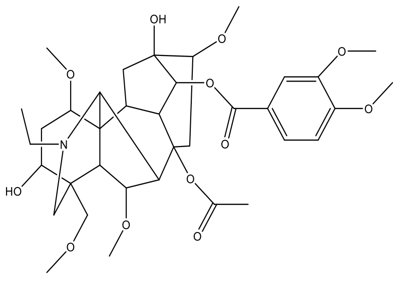

Pseudoaconitine (42)

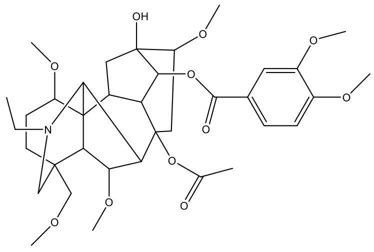

Bikhaconitine (47)

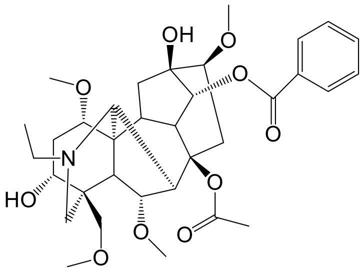

Indaconitine (48)

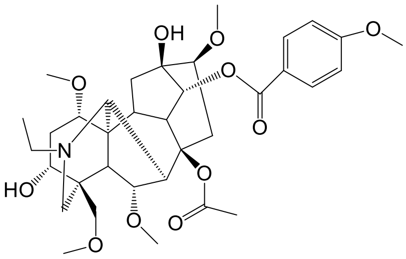

Yunaconitine (49)

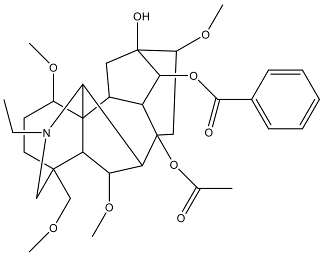

Chasmaconitine (53)

Figure S3. The structures of DDAs identified in the tincture

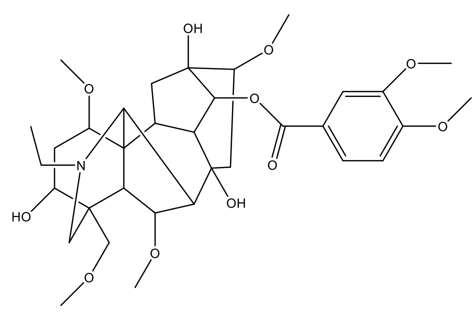

8- Deacetylseudoaconitine (32)

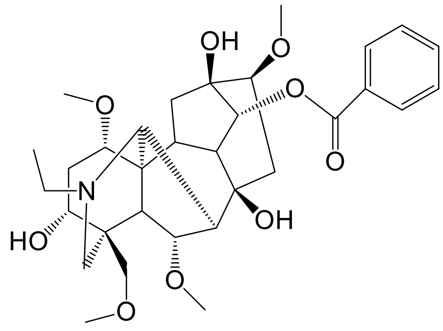

Ludaconitine (34)

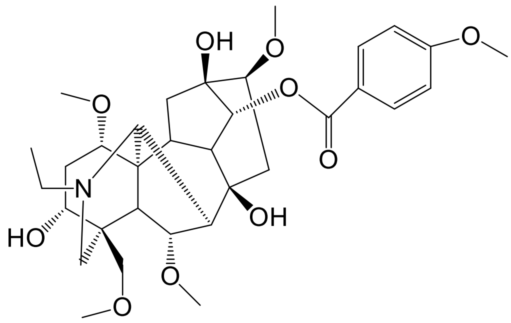

8-Deacetylyunaconitine (36)

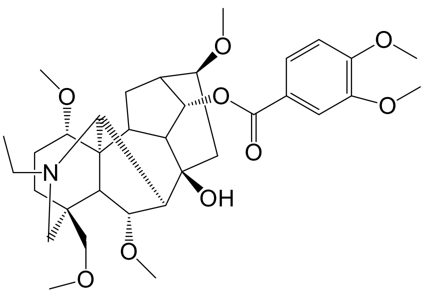

Falconeridine (45)

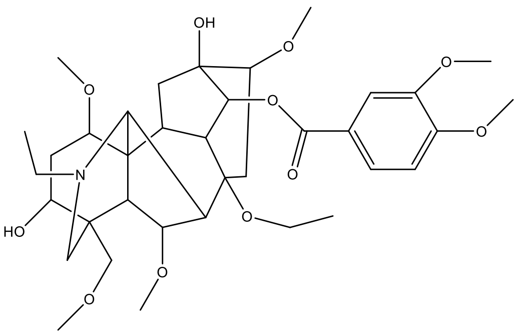

3′-Methoxyacoforestinine (46)

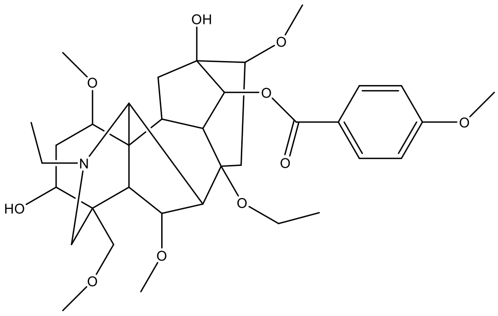

Acoforestinine (56)

Figure S4. The structures of MDAs identified in the tinctur

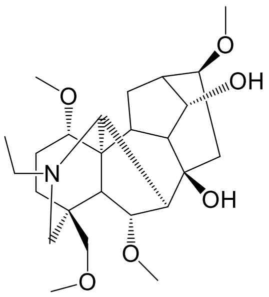

10-OH-neoline (5)

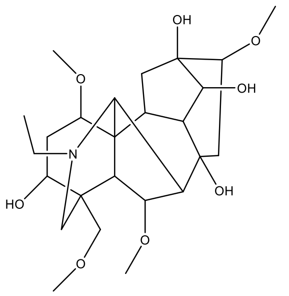

Pseudoaconine (7)

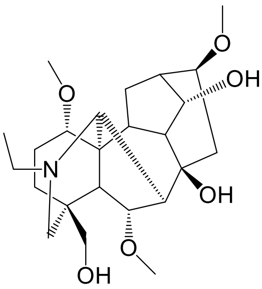

Neoline (11)

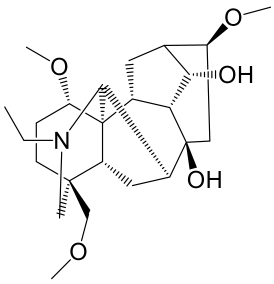

Talatisamine (14)

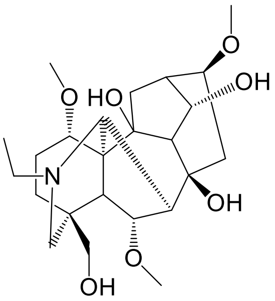

Chasmanine (18)

Figure S5. The structures of ADAs identified in the tincture

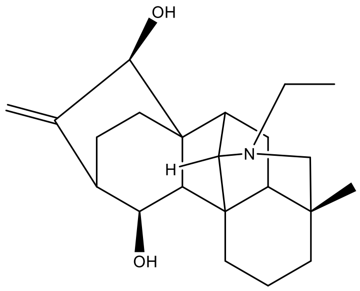

Guan-fu Base H (15)

Figure S6. The structure of C20-diterpenoid alkaloids identified in the tincture

Table S1. Retention times of *Aconitum* alkaloids in the tincture on an HSS T3 column.

Compound	Molecular Formula	m/z calculated $[M+H]^+$	RT(min)*	Class	Identification
1	$C_{23}H_{35}NO_6$	422.2537	1.57	ADA	
2	$C_{23}H_{37}NO_6$	424.2694	1.83	ADA	
3	$C_{23}H_{37}NO_5$	408.2744	2.21	ADA	
4	$C_{23}H_{37}NO_6$	424.2694	2.28	ADA	
5	$C_{24}H_{39}NO_7$	454.2799	2.27	ADA	10-OH-neoline
6	$C_{22}H_{33}NO_3$	360.2533	2.40	C20	
7	$C_{25}H_{41}NO_8$	484.2905	2.46	ADA	pseudoaconine
8	$C_{22}H_{33}NO_3$	360.2533	3.26	unidentified	
9	$C_{23}H_{33}NO_5$	404.2431	3.40	unidentified	
10	$C_{20}H_{23}NO_4$	342.1700	4.00	unidentified	
11	$C_{24}H_{39}NO_6$	438.2850	3.80	ADA	neoline
12	$C_{25}H_{41}NO_7$	468.2956	3.97	ADA	
13	$C_{27}H_{43}NO_9$	526.3011	3.95	ADA	
14	$C_{24}H_{39}NO_5$	422.2901	4.72	ADA	talatisamine
15	$C_{22}H_{33}NO_2$	344.2584	5.06	C20	guan-fu Base H
16	$C_{26}H_{41}NO_8$	496.2905	5.35	ADA	
17	$C_{26}H_{41}NO_7$	480.2956	5.35	ADA	
18	$C_{25}H_{41}NO_6$	452.3007	5.46	ADA	chasmanine
19	$C_{23}H_{31}NO_5$	402.2275	5.56	MDA	
20	$C_{27}H_{45}NO_7$	496.3269	5.69	ADA	
21	$C_{27}H_{43}NO_7$	494.3112	5.78	DDA	
22	$C_{29}H_{45}NO_{10}$	568.3116	5.86	MDA	
23	$C_{23}H_{31}NO_3$	370.2377	5.94	unidentified	

Compound	Molecular Formula	m/z calculated $[M+H]^+$	RT(min)*	Class	Identification
24	$C_{27}H_{43}NO_8$	510.3061	5.99	ADA	
25	$C_{27}H_{43}NO_7$	494.3112	6.01	MDA	
26	$C_{33}H_{47}NO_{11}$	634.3222	6.00	MDA	
27	$C_{27}H_{45}NO_6$	480.3320	6.17	ADA	
28	$C_{40}H_{53}NO_{16}$	804.3437	6.16	unidentified	
29	$C_{29}H_{47}NO_9$	554.3324	6.28	ADA	
30	$C_{33}H_{47}NO_{10}$	618.3273	6.42	MDA	
31	$C_{38}H_{55}NO_{13}$	734.3746	6.42	unidentified	
32	$C_{34}H_{49}NO_{11}$	648.3378	6.47	MDA	8-deacetylpseudoaconitine
33	$C_{35}H_{49}NO_{12}$	676.3328	6.54	DDA	
34	$C_{32}H_{45}NO_9$	588.3167	6.71	MDA	ludaconitine
35	$C_{35}H_{49}NO_{11}$	660.3378	6.82	DDA	
36	$C_{33}H_{47}NO_{10}$	618.3273	6.83	MDA	8-deacetylyunaconitine
37	$C_{35}H_{51}NO_{11}$	662.3535	6.91	MDA	
38	$C_{34}H_{49}NO_{10}$	632.3429	6.99	MDA	
39	$C_{36}H_{51}NO_{12}$	690.3484	6.94	DDA	pseudoaconitine isomer
40	$C_{35}H_{49}NO_{11}$	660.3378	7.08	DDA	
41	$C_{33}H_{45}NO_9$	600.3167	7.17	DDA	delphinine
42	$C_{36}H_{51}NO_{12}$	690.3484	7.20	DDA	pseudoaconitine
43	$C_{32}H_{45}NO_7$	556.3269	7.42	MDA	
44	$C_{29}H_{33}NO_5$	476.2431	7.43	MDA	
45	$C_{34}H_{49}NO_9$	616.3480	7.59	MDA	falconeridine
46	$C_{36}H_{53}NO_{11}$	676.3691	7.62	MDA	3′-methoxyacoforestinine
47	$C_{36}H_{51}NO_{11}$	674.3535	7.67	DDA	bikhaconitine
48	$C_{34}H_{47}NO_{10}$	630.3273	7.71	DDA	indaconitine
49	$C_{35}H_{49}NO_{11}$	660.3378	7.73	DDA	yunaconitine
50	$C_{36}H_{49}NO_{12}$	688.3328	7.75	DDA	
51	$C_{38}H_{55}NO_{12}$	718.3797	8.05	unidentified	
52	$C_{36}H_{53}NO_{10}$	660.3742	8.21	MDA	
53	$C_{34}H_{47}NO_9$	614.3324	8.18	DDA	chasmaconitine
54	$C_{36}H_{51}NO_{10}$	658.3586	8.33	DDA	
55	$C_{35}H_{51}NO_{10}$	646.3586	8.18	MDA	acoforestinine(8-O-etylyunaconitine)
56	$C_{34}H_{49}NO_9$	616.3480	8.25	MDA	
57	$C_{35}H_{49}NO_{10}$	644.3429	8.79	DDA	crassuline A isomer
58	$C_{34}H_{47}NO_8$	598.3374	8.88	DDA	

Note: * The RT was obtained using a Waters ACQUITY HSS T3 (150 × 2.1 mm, 1.7 μm). All other mass spectrometry and chromatography methods were identical to those described in the article.
